# The protective role of vitamin E on the testicular tissue in rats exposed to sodium arsenite during the prenatal stage till sex maturity: A stereological analysis

**Published:** 2012-11

**Authors:** Malek Soleimani Mehranjani, Rezvan Taefi

**Affiliations:** *Department of Biology, Faculty of Sciences, Arak University, Arak, Iran.*

**Keywords:** *Sodium arsenite*, *Vitamin E*, *Stereology*, *Testis*, *Rat*

## Abstract

**Background: **Vitamin E is an effective antioxidant, protecting cells against oxidative stress.

**Objective:** In this investigation the protective effect of vitamin E on the testis during development and spermatogenesis in rats exposed to sodium arsenite was evaluated.

**Materials and Methods:** Pregnant Wistar rats were divided into 4 groups (n=8) control, sodium arsenite (8 mg/kg/day), sodium arsenite+vitamin E (100 mg/kg/day) and vitamin E. Treatment was carried out from day seven of pregnancy till 90 days. Finally the right testis was stereologically studied. The obtained data was analyzed using one way ANOVA and Tukey's test and the means difference was considered significant at p<0.05.

**Results:** The weight and volume of testis, volume of seminiferous tubules and its diameter, volume of interstitial tissue, height of germinal epithelium and the total number of types A and B spermatogonia, spermatocyte, spermatid and sertoli cells reduced significantly in sodium arsenite group compared to the control. Co-administration of vitamin E and sodium arsenite compensated the adverse effects of sodium arsenite on the above parameters.

**Conclusion:** We concluded co-treatment of rats with sodium arsenite and vitamin E could prevent the adverse effects of sodium arsenite exposure on the testicular tissue during the prenatal stage till sex maturity.

## Introduction

Arsenic as an environmental toxicant is used in the production of agricultural pesticides, herbicides, insecticides, rodenticides, food and wood preservatives, metallurgic applications, glass production, as a catalyst in several manufacturing process and in medicine. Human are exposed to arsenic mainly through water, food and drugs. The main source of environmental arsenic exposure in most populations is the drinking water (1-3). 

Arsenic exposure causes both acute and chronic disorders in human, such as diabetes, gastrointestinal tract disorders, degenerative, inflammatory, and neoplastic changes of the respiratory, hematopoietic, cardiovascular, and nervous systems (4-6). Investigations have also shown that arsenite has toxic effects on the male reproductive system, accumulating in the testes, seminal vesicle and the prostate glands, characterized by low sperm count, decrease in sperm motility and increase in abnormal spermatozoa, leading to genotoxicity in testicular tissue and impairment of spermatogenesis (1, 7-9). 

In addition, the effects of postnatal sodium arsenite exposure on Leydig cells of Wistar rats testes has been studied, revealing a significant decrease in testicular weight and the total number of Leydig cells at postnatal day 21 in the treated rats (10). Since Leydig cells of the testis are responsible for the biosynthesis and secretion of androgens, arsenite toxicity may disturb the development of the male reproductive system (11).

Arsenic can also cross the placenta. This experimental study investigate the reproductive and developmental toxicity of prenatal arsenic exposure in rats (12, 13). Oral treatment of inorganic arsenic at maternally toxic doses can reduce fetal body weight affecting fetal development (14). Arsenic toxicity is due to the fact that arsenic increases the formation of free radicals inducing a cellular redox imbalance leading to oxidative stress, which in turn initiates harmful reactions altering the membrane structure and cellular integrity through lipid peroxidation leading to irreversible cell damages (15).

1. Vitamin E (α-tocopherol) is a naturally occurring antioxidant that plays an important role by inactivating harmful free radicals produced through normal cellular activity and from various stressors thus terminating lipid peroxidation and stabilizing the molecular composition of cellular membranes, preventing the harmful effects of reactive oxygen species (ROS). Therefore vitamin E is used to ameliorate the toxic effects of arsenic (16-20).
2. The present study was designed to investigate the toxic effect of sodium arsenite treatment on the testicular tissue of rats from prenatal to maturation for a period of 90 days. Since vitamin E is vital for normal reproduction and testicular function, therefore, the preventing role of vitamin E, as a strong antioxidant, on the undesired effects of sodium arsenite on the testis was also evaluated in rats co-treated with sodium arsenite and vitamin E using stereological techniques (21, 22).

## Materials and methods

**Animals and treatments**

Male and female Wistar rats with average weight of 200±25g were purchased from Pasteur institute, Iran and kept in the animal house of Arak University under standard condition of 21±2^o^C, 12h light - 12h dark and enough food and water. 

After mating, based on vaginal plug observation, female pregnant rats were divided into 4 groups (n=8), including control, sodium arsenite (8mg kg^-1^ per day) (Merk company, Germany), vitamin E (100mg kg^-1^ per day) (Sigma Aldrich, Steinheim, USA) and sodium arsenite + vitamin E. 

The treatment was carried out from day 7 of pregnancy to day 21 of postnatal, through mother's milk. After weaning, the male pups (F1 generation) were divided into the same groups (n=8) as their parents, and the same treatments were continued orally till maturation (90 days) (23). The experiments were approved by the local ethical committee at Arak University.

**Chemicals and dosage**

All the chemicals in this study were purchased from Merck Company, Hohenbrunn, Germany unless it is mentioned elsewhere. 

The sodium arsenite dose was selected on the basis of the doses that have been used in the previous investigations with pathological consequences on male reproductive systems of rats and the same logic was also considered for vitamin E dose (19, 24-26). The duration of exposure to sodium arsenite was 90 days, which is the required time for completion of maturation, and vitamin E treatment was also done along with sodium arsenite.

**Tissue preparation**

At the end of the treatment, the rats were weighed and anesthetized using diethyl ether, then they killed were, and their right testis were taken out and weighed. The volume of the testis was estimated using the immersion method and then fixed in the modified Davidson^'^s fluid fixative **(**27**, **28**)**.

**Stereological study**

The orientator method was used to obtain isotropic uniform random (IUR) sections (29, 30). For this purpose, testis was randomly placed on the φ clock, which is divided into nine equal parts. By choosing a random number from one to nine, an appropriate cut was made along the selected number, which resulted in two pieces of testis. The first piece was then placed on the θ clock- which divided into nine unequal parts-along with its cut surface on the 0-0 axis, then random number was selected again and a parallel cut was made along the selected number. 

The other piece that resulted from the cut made on the φ clock was placed on the θ clock vertically so that its cut surface overlapped the 0-0 axis. This piece was also cut parallel along a randomly selected number. After tissue processing, sections of 5 and 20μm thickness were cut using a microtome and stained using Heidenhain Azan method.

**Estimating the shrinkage and the total volume of testis**

To estimate the shrinkage, three random segments were prepared using trocar from IUR sections. For each segment the two vertical diameters were measured and their mean radius was estimated and considered as pre-fixing radius (r_before_). 

After fixation, tissue processing and sectioning, staining was carried out and the same measurements were performed as above, and the obtained mean radius was considered as the post-fixing radius (r_after_). Using the following equitation, the amount of shrinkage in each testis was estimated (29).

-Shrinkage=1-rafter 2rbefor 232

To obtain the true volume of testis the amount of shrinkage was subtracted from the volume estimated by the immersion method.

**Estimating the volume of seminiferous tubules and interstitial tissue**

To estimate the volume of seminiferous tubules and interstitial tissue, using the systematic random sampling method, an average of 5-7 fields per each 5μm section was evaluated by randomly placing the point probe on each field. The total points superimposed on the whole field 

$\left(\sum_{i=1}^{m}P_{\mathrm{total}}\right)$

along with the total points superimposed on the seminiferous tubules 

$\left(\sum_{i=1}^{m}P_{\mathrm{tubules}}\right)$

and the total points superimposed on the interstitial tissue 

∑i=1mPint erstitial

were counted, and volume density of each was estimated using the following equation:

$V_{V}=\frac{\sum_{i=1}^{n}P_{\left(x\right)}}{\sum_{i=1}^{n}P_{\mathrm{total}}}$

where x is the interstitial tissue or seminiferous tubules. The total volume of each compartment was also calculated by multiplying its volume density (Vv) by the total volume of the testis.

**Estimating the length, diameter, height of the germinal epithelium and the basement membrane thickness of seminiferous tubule**

To estimate the length of seminiferous tubules, 5-7 random fields from 5 μm thick sections, using the objective of ×10, were selected and the number of the selected seminiferous tubules profiles was counted using an unbiased counting frame (30-31), thus, an average of 110-130 seminiferous tubules per testis was estimated. The length density of seminiferous tubules was also estimated using following formula: 

LV=2×∑i=1mQiaf. ∑i=1mpi

where ΣQi is the total number of the selected tubules, a/f for frame area in tissue scale and ΣPi is the total points superimposed on the testis tissue. To obtain the absolute length of the seminiferous tubules, the lengths density (Lv) was multiplied by the total volume of the testis.

To estimate the mean diameter of seminiferous tubule, we used the Olympus DP12 microscope equipped with camera. The diameter of the tubules was measured on the sampled tubules in the counting frame used for estimating the length of the seminiferous tubules. The diameter was measured perpendicularly to the long axis of the tubules where the tubules are widest (31). Approximately 110-130 tubules were measured. To estimate the height of the germinal epithelium the following equation was used

$H=\frac{V_{V}}{S_{V}}$

in which Vv is the volume density of the germinal epithelium and Sv is the surface density of the germinal epithelium. For this purpose, an average of 8 to 10 fields, using the objective of ×10, from all of the 5μm thick sections of rat testis were studied using the systematic random sampling method.

To obtain the volume density of the germinal epithelium, the total number of points of the point probe superimposed on each image of the testis was counted and then the total number of points superimposed on the germinal epithelium was also counted and divided into the total number of points counted for the testis.

To estimate the surface density (Sv) of the germinal epithelium, the total number of points superimposed on the germinal epithelium of the seminiferous tubule (ΣPi), the length of the linear test probe in actual tissue scale (L/P), along with the total number of intersections of linear test probe with the inner surface of the germinal epithelium (ΣIi) were counted. The surface density (Sv) was then estimated using the following equation:

SV=2×∑i=1nIiLp×∑i=1npi

To estimate the mean basement membrane thickness of seminiferous tubules, the harmonic mean of basement membrane thickness was estimated (17). A number of the fields was selected from all of the 5μm thick sections using the objective of ×100, then a probe consists of isotropic lines was superimposed on the images and the distance between the inner and outer surface of basement membrane was measured by drawing a line from the outer surface to the touch point of the isotropic line with the inner surface of the membrane. 

The distance of the drawn line was considered as the thickness of basement membrane. An average of 110-120 intercepts was estimated, and the mean basement membrane thickness was calculated using the following equation:

Harmonic mean layer thickness= 8/3π × Harmonic mean of orthogonal intercepts where harmonic mean= number of measurements/ sum of the reciprocal of orthogonal intercepts (oi) lengths =

$\mathrm{number of measurement}/(\frac{1}{O_{i_{1}}}+\frac{1}{O_{i_{2}}}+\frac{1}{O_{i_{3}}}+\frac{1}{O_{i_{4}}}+\ldots )$

**Estimating the number of spermatogonia (A and B), spermatocyte, spermatid and sertoli cells**

To estimate the number of cells, the optical dissector method and the unbiased counting frame were used (19, 21). A number of fields, using the objective of ×100, from all of the 20μm thick sections were selected and a microcator (ND 221 B, Heidenhain, Germany) was used for counting. Using the following equation, the number density (Nv) of different types of cells was estimated.

Nv=∑i=1nQih×∑n=1npi×af

Where ΣQi is the total number of the counted cells, h is the tissue thickness considered for counting, a/f is the frame area in true tissue scale and ΣPi is the total number of points superimposed on the selected fields. The result of the equation was then multiplied by the total volume of testis to obtain the total number of cells (Ntotal = Nv×Vtotal).

**Statistical analysis**

The results were analyzed by one–way analysis of variance (ANOVA) and Tukey’s test using the SPSS V11/0 software. The means were considered significantly different at p<0.05. 

## Results

**Histopathological findings**

**Figure 1:**
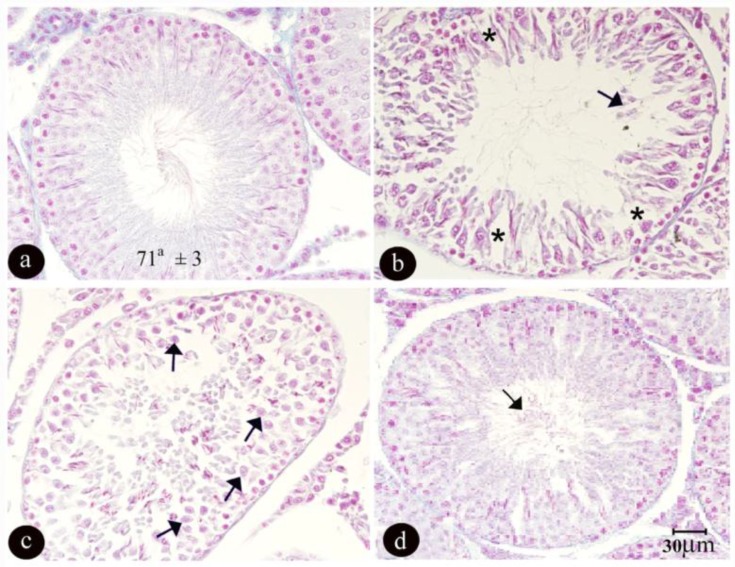
Micrographs of testis tissue in different groups of rats after 90 days of treatment: a) the control group, showing the normal amount of sperm and a germinal epithelium with normal height and regular arrangement. b and c) the sodium arsenite group (8 mg/kg/day), showing reduction in the amount of sperm with an irregular and vacuolated (stars) germinal epithelium and the formation of giant cells (arrows) due to the degeneration of the germinal epithelium. d) the sodium arsenite + vitamin E (100 mg/kg/day) group, showing an increase in the amount of sperm (arrow) with a more regular arrangement of germinal epithelium compared with the sodium arsenite group. (Sections with 5μm thickness and Heidenhain Azan staining).

An irregular vacuolated germinal epithelium with a reduction in height was observed in the sodium arsenite treated group. Giant cells were seen following germinal epithelium degeneration in the mentioned group. The amount of sperm also seemed to be less in this group compare to the other groups (Figure 1).

**The total volume of testis, seminiferous tubules and interstitial tissue**

A highly significant reduction in the mean total volume of testis and seminiferous tubules was observed in the sodium arsenite group compared to the control ones (p<0.01). While the sodium arsenite + vitamin E group showed no significant reduction in the mentioned parameters compared to the control group (p>0.05). Comparing the mean volume of the interstitial tissue showed a significant reduction in the sodium arsenite group relative to the control group (p<0.01). The same result was also obtained for the sodium arsenite + vitamin E group (Table I).

**The length, diameter, basement membrane thickness and the germinal epithelium height of seminiferous tubules**

The sodium arsenite group showed a highly significant reduction in the mean diameter and the height of the germinal epithelium of the seminiferous tubules compare to the control group (p<0.001). While the sodium arsenite+ vitamin E group did not show a significant difference in the above parameters when compared to the control group (p>0.05). 

Sole treatment with vitamin E lead to a significant increase (p<0.01) in the mean diameter, basement membrane thickness and the height of the germinal epithelium of seminiferous tubules compared to the other groups. Meanwhile the length of the seminiferous tubules showed no significant difference (p>0.05) in all four groups (Table II).

**Number of the spermatogonia cells (A and B), spermatocyte, spermatid and sertoli cells**

A significant reduction in the mean number of spermatocyte, spermatid and sertoli cells was seen in the sodium arsenite group compared to the control rats (p<0.05). While the sodium arsenite + vitamin E group did not show a significant difference in the above parameters when compared to the control group (p>0.05), except for the mean number of spermatids which didn't increase to the normal level in this group (p<0.05). Sole treatment with vitamin E lead to a significant (p<0.01) increase in the mean number of spermatogonia cells (A and B) and spermatocytes compared to the other groups (Table III).

**Body and testis weight**

At the end of weaning, no significant difference was found in the mean body weight of the four groups. While, the mean body weight in the sodium arsenite group reduced significantly (p<0.001) compared to other groups at the end of the treatment. Co-treatment of vitamin E + sodium arsenite could compensate the body weight reduction observed in the sodium arsenite group (p>0.05). Rats treated with sodium arsenite showed a significant reduction in the testis weight when compared to the control group. However, the application of sodium arsenite + vitamin E was not able to ameliorate the reduction in the testis weight compared with the sodium arsenite group (Table IV).

**Table I:** Total volume of testis (mm^3^), seminiferous tubules and interstitial tissue (mm^3^)

**Groups**	**Testis volume**	**Seminiferous tubules volume**	**Interstitial tissue volume**
Control	1238^ a^ ± 91	1011^ a^ ± 77	227^a^ ± 23
Sodium arsenite	1033^ b^ ± 51	87^ b^ ± 56	141^b^ ± 19
Vitamin E	1123^ a b^ ± 94	978^ a b^ ±92	145 .5^ b^ ± 13
Sodium arsenite + vitamin E	1112^ a b^ ± 81	978^ a b^ ± 63	134 ^b^ ± 30

Values are means±SD.

Means with the same letter do not differ significantly from each other (ANOVA, Tukey's test, and p > 0.05).

**Table II:** Mean length, diameter, basement membrane thickness and germinal epithelium height of the testis somniferous tubules (mm^3^)

**Groups**	**Length of seminiferous tubules (m)**	**Diameter of seminiferous tubules ** **)** **µm** **(**	**Thickness of basement membrane ** **)** **µm** **(**	**Height of germinal** **epithelium (µm)**
Control	16^ a^ ± 4	268^ a^ ± 5	8.4^ a^ ± 0.2	71^ a^ ± 3
Sodium arsenite	15^ a^ ± 3	254.5 ^b^ ± 5	8.7 ^ a^ ± 0.2	58 ^ b^ ± 2
Vitamin E	13.5^ a^ ± 2	287^ c^ ± 6.5	9.3 ^ b^ ± 0.4	90^ c^ ± 6
Sodium arsenite+vitamin E	12.5^ a^ ± 2	272 ^ a^ ± 4.5	8.6 ^ a^ ± 0.02	75^ a^ ± 6

Values are means ± SD.

Means with the same letter do not differ significantly from each other (ANOVA, Tukey's test, and p > 0.05).

**Table III:** Number of the spermatogonia (A and B), spermatocyte, spermatid and sertoli cells

**Groups**	**Spermatogonia A** **10** ^6^	**Spermatogonia B** **10** ^6^	**Spermatocyte** **10** ^6^	**Spermatid** **10** ^6^	**Sertoli cells** **10** ^6^
Control	8.7 ^a^ ± 0. 4	1.8^ a^ ± 1	139^ a^ ± 13^ a^	436 ^a^ ± 26	31^a^ ± 3
Sodium arsenite	6.2 ^b^ ± 1.4	1.2^ b^ ± 2	101^ b^ ± 10	267^ b^ ± 34	21^b^ ± 3
Vitamin E	10.8^ c^ ± 0.9	2.7^ c^ ± 5	171^ c^ ± 14	497^ a^ ± 46	37^ ac^ ± 5
Sodium arsenite + vitamin E	8.9^ a^ ± 0.8	1.8^ a^ ± 2	135^ a^ ± 14	310^ b^ ± 46	28^ ad^ ± 2

Values are means ± S.D.

Means with the same letter do not differ significantly from each other (ANOVA, Tukey's test, and p > 0.05).

**Table IV:** Testis and body weight (g)

**Groups **	**Testis weight**	**Body weight at the end of weaning**	**Body weight at the end of treatment**
Control	1.5^ a^ ± 0.85	62 ^ a^ ± 4	373 ^ a^ ± 17
Sodium arsenite	1.4^ b^ ± 0.36	60 ^ a^ ± 2	306 ^ b^ ± 7
Vitamin E	1.5 ^ab^ ± 0.71	58 ^ a^ ± 3	374^ ac^ ± 8
Sodium arsenite + vitamin E	1.4^ ab^ ± 0.49	59^ a^ ± 5	356^ ad^ ± 6

Values are means ± S.D.

Means with the same letter do not differ significantly from each other (ANOVA, Tukey's test, and p > 0.05).

## Discussion

Our results showed that treatment with sodium arsenite caused a significant reduction in the mean total volume of testis and the volume, diameter and the germinal epithelium height of the seminiferous tubules. Other studies have also indicated that treatment with sodium arsenite causes shrinkage, atrophy and diameter reduction in the seminiferous tubules and a significant reduction in the volume of testis (9, 32-35). 

Since the seminiferous tubules are the main component of the testis, it can be deduced that testis volume reduction may be due to the reduction in the volume of seminiferous tubules. Treatment with sodium arsenite also lead to a significant decrease in the number of spermatogonia type A and B, spermatids and sertoli cells compared to the control group. Other studies have also indicated that sodium arsenite can disturb spermatogenesis. 

Ahmad *et al* reported a reduction in the number of spermatid and spermatozoa cells and also damage to the Leydig and sertoli cells following administration of 6mg/kg of sodium arsenite in rats (32). In addition Sarkar and co-workers, obtained the same results during the treatment of adult mice with sodium arsenite 30 and 40mg/L through drinking water for a period of 30, 45 and 60 days (34). 

Since Leydig cells play an important role in the function and structure of seminiferous tubules and synthesis of testosterone which is vital for the regulation of spermatogenesis, decrease in the number of Leydig cells can be considered as a disturbing factor in the process of spermatogenesis. 

On the other hand sodium arsenite can elevate the serum corticosterone level which in turn reduces the serum gonadotrophin, FSH and LH levels, which is required for the initiation and maintenance of spermatogenesis (22, 36, 37). Therefore the reduction in the number of germinal cells could be due to the imbalance of hypothalamus-hypophysis axis hormones. Studies have reported that treatment with sodium arsenite causes a reduction in the activity of scavenger enzymes such as glutathione reductase and superoxide dismutase leading to oxidative stress in sertoli cells (33, 35, 38-39). 

Free radicals such as ROS disturb cell membrane integrity through lipid peroxidation which causes cell damage. Since sertoli cells support germ cells by the secretion of proteins such as core protein Histon, Androgen Binding Protein (ABP), ABP-heat shock protein 27, N-cadherin and desmoglein, therefore damage to sertoli cells can alter the structural and functional activity of these proteins which may be another reason in the reduction of the number of germ cells (31). 

Furthermore, changes in the enzymes of testis following exposure to sodium arsenite is also involved in the damage of testicular tissue. In this case Pant *et al* (2004) reported the alterations in the activity of testicular enzymes including sorbitol dehydrogenase, acid phosphatase and lactate dehydrogenase in mice treated with 53.39mmol/L of sodium arsenite, which can damage the germ cells. 

They also found an increase in *γ-GT* activity, a sertoli cells marker, which may be as a result of arsenite interference with the normal physiology of sertoli cells, influencing the development of spermatocytes and spermatids (1).

In the present study, sodium arsenite lead to a significant body weight loss in rats at the end of the treatment period. Other studies have also reported a significant reduction in the body weight of rats following exposure to sodium arsenite with doses of 5 and 6 mg/kg. (32, 40-41). This is possibly due to the fact that sodium arsenite decreases appetite in rats as a consequence of its toxicity. 

As the present results showed co-treatment of rats with sodium arsenite and vitamin- E compensated the reduction of body weight, testis weight and volume, seminiferous tubules volume, diameter and its germinal epithelium height, and also the reduction in the number of germinal cells to the control level. It is believed that the reduction in the above parameters may be relevant to the oxidative stress induced following treatment with sodium arsenite, which is an important factor in explaining arsenic toxicity, influenced by its oxidation state, which has a high affinity for the membrane sulfhydryl groups, resulting in lipid peroxidation (38, 42-44). 

Vitamin E, a strong lipid soluble antioxidant present in the cell, naturally accumulates in the membranes of mitochondria and endoplasmic reticulum and protects testicular cells from lipid peroxidation. In this case Gavazza *et al* (2001) showed that treatment of rats with a single dose of 100mg/kg of alpha-tocopherol could protect testis mitochondria from lipid peroxidation (45). 

In addition, rats treated with 25mg/kg of vitamin E compared with the rats treated only with sodium arsenite indicated an increase in the antioxidant enzymes activity and glutathione (GSH) concentration and decrease in lipid peroxidation (38). In this case, vitamin E scavenges free radicals in order to preserve cell membrane functions such as ion transport and membrane fluidity through maintaining the sulfhydryl groups of membrane proteins, potentially decreasing the rate of lipid peroxidation (38,42). 

The obtained desirable changes in the testicular tissue following treatment with vitamin E could be due to the fact that vitamin E (alpha-tocopherol) can interfere with the reactions that lead to lipid peroxidation, compensating for the sodium arsenite induced toxicity (45).

## Conclusion

In conclusion, according to our results, vitamin E, as a strong antioxidant, could compensate for the majority of the undesirable changes due to sodium arsenite exposure in the rat testis structure. 

In addition, sole treatment with vitamin E could significantly increase the seminiferous tubules diameter and basement membrane thickness, height of germinal epithelium as well as the number of types A and B spermatogonia and spermatocytes. Therefore, consumption of vitamin E in the case of sodium arsenite toxicity is recommended. 
